# Inflammaging and Complement System: A Link Between Acute Kidney Injury and Chronic Graft Damage

**DOI:** 10.3389/fimmu.2020.00734

**Published:** 2020-05-07

**Authors:** Rossana Franzin, Alessandra Stasi, Marco Fiorentino, Giovanni Stallone, Vincenzo Cantaluppi, Loreto Gesualdo, Giuseppe Castellano

**Affiliations:** ^1^Nephrology, Dialysis and Transplantation Unit, Department of Emergency and Organ Transplantation, University of Bari Aldo Moro, Bari, Italy; ^2^Department Translational Medicine, University of Piemonte Orientale, Novara, Italy; ^3^Nephrology, Dialysis and Transplantation Unit, Department of Medical and Surgical Sciences, University of Foggia, Foggia, Italy

**Keywords:** renal aging, complement system, AKI-to-CKD transition, cellular senescence and SASP, complement inhibition therapy

## Abstract

The aberrant activation of complement system in several kidney diseases suggests that this pillar of innate immunity has a critical role in the pathophysiology of renal damage of different etiologies. A growing body of experimental evidence indicates that complement activation contributes to the pathogenesis of acute kidney injury (AKI) such as delayed graft function (DGF) in transplant patients. AKI is characterized by the rapid loss of the kidney’s excretory function and is a complex syndrome currently lacking a specific medical treatment to arrest or attenuate progression in chronic kidney disease (CKD). Recent evidence suggests that independently from the initial trigger (i.e., sepsis or ischemia/reperfusions injury), an episode of AKI is strongly associated with an increased risk of subsequent CKD. The AKI-to-CKD transition may involve a wide range of mechanisms including scar-forming myofibroblasts generated from different sources, microvascular rarefaction, mitochondrial dysfunction, or cell cycle arrest by the involvement of epigenetic, gene, and protein alterations leading to common final signaling pathways [i.e., transforming growth factor beta (TGF-β), p16^*ink*4*a*^, Wnt/β-catenin pathway] involved in renal aging. Research in recent years has revealed that several stressors or complications such as rejection after renal transplantation can lead to accelerated renal aging with detrimental effects with the establishment of chronic proinflammatory cellular phenotypes within the kidney. Despite a greater understanding of these mechanisms, the role of complement system in the context of the AKI-to-CKD transition and renal inflammaging is still poorly explored. The purpose of this review is to summarize recent findings describing the role of complement in AKI-to-CKD transition. We will also address how and when complement inhibitors might be used to prevent AKI and CKD progression, therefore improving graft function.

## Overview of the Complement System

Complement is an essential part of the innate immune system. Over a century ago, complement was first identified by Paul Ehrilch as a heat-labile component in serum that literally “complemented” the antibody- and cell-mediated immune responses against pathogens (1). Today, we do know that complement system consists of more than 40 blood-circulating, membrane-associated, and intracellular proteins. Complement can be activated in the serum, in local tissue, and at intracellular level (2) and exerts three major physiological functions. First, complement proteins are involved in host defense against infection (3). This activity is mediated by several events: (i) the pathogens opsonization (i.e., covalent C3b, C3d, C4b complement fragments deposition on microbial surfaces that boost phagocytosis), (ii) the leukocytes chemotaxis and activation that amplify the inflammatory process (i.e., the binding of complement anaphylatoxin to receptors on leukocytes), and (iii) the direct lysis of bacteria or infected cells. Second, complement can be considered as a connection between innate and adaptive immune response (4). Indeed, the C1q, the principal component of the classical pathway, can activate complement cascade after the binding to antibody–antigen complexes, which originated during the adaptive immune response. In addition, complement can also enhance the antibody response and consolidate the immunological memory since C3 receptors are expressed on B cells, antigen-presenting cells (APC), and follicular dendritic cells (5). Third, after the resolution of inflammatory injury, complement mediates the clearance of apoptotic/necrotic, ischemic, or damaged self-cells (i.e., by the binding of C1q or C3 fragments to host self-surfaces) (6).

**FIGURE 1:**
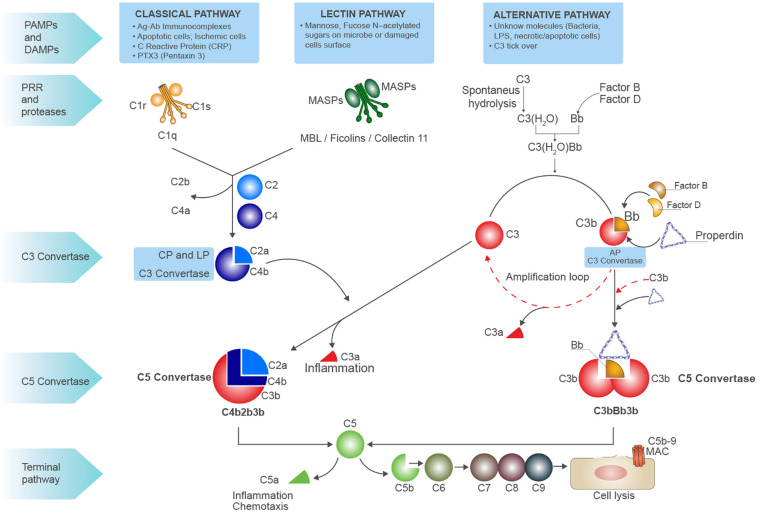
Schematic overview of complement system. Complement system can be initiated by three different pathways: the classic pathway, the lectin pathway, and the alternative pathway, all converging to the formation of C3 convertases. The classic pathway is initiated by the binding of C1q globular domains to the Fc of immunoglobins bound to their antigen (immunocomplexes), apoptotic or ischemic cells, acute phase proteins [i.e., C-reactive protein (CRP) and Pentraxins]. When the binding of C1q to substrate occurs, a conformational change of C1q leads to activation of proteases C1r and C1s that are associated to C1q. It activates C1s, then cleaves C4 into C4b, subsequently C2 is cleaved which binds to C4b forming the CP (membrane-attached) C3 convertase, the C4b2a complex. This classical C3 convertase activates and cleaves C3 molecules to C3b and C3a. The pattern recognition receptor (PRR) of lectin pathway involves several molecules as MBL, Ficolins, and Collectin-11 that, after binding to mannose, fucose, or *N*-acetylated residues on microbial surfaces or damaged cells, can activate the serine proteases MASP1 and MASP2 leading to C3 convertase formation as for CP. At low level, the activation of alternative pathway (AP) can be induced by spontaneous hydrolysis of C3 into C3(H_2_O), an event called C3 tick-over. The hydrolysis changes the structure of C3 by the translocation of the thiol ester domain that allows the new formed structure to form covalent bonds with -OH or -NH2 residues on the target surfaces. The C3(H_2_O) can bind factor B (FB), resulting in the cleavage of FB by factor D (FD) and generating Ba and Bb and the formation of the AP C3 convertase C3(H_2_O)Bb. The C3(H_2_O)Bb complex is the initial C3 convertase of the AP (fluid phase C3 convertase) and can cleave C3 to C3a and C3b. The C3b fragment can bind to FB, and after the cleavage of FB by FD, the C3 convertase C3bBb (high level) is formed. This C3 convertase cleaves more C3 to C3b to generate even more C3 convertase in an amplification loop. The protein properdin stabilizes C3bBb. After formation of the classical C3 convertase C4b2a or the alternative C3 convertase C3bBb, the final pathway (common to all three pathways) may be initiated. An additional C3b molecule is incorporated in both the C3 convertases leading to the formation of the C5 convertase. Properdin stabilization occurs in AP C5 convertase formation (C3bBb3b). The C5 convertase cleavages C5 into C5a (the anaphylatoxin) and C5b, C5b then binds to C6, and this allow the binding of C7, C8, and C9 and results in the formation of the C5b-9 terminal membrane attack complex (MAC). The latter forms pores in the membrane of pathogens and damaged self-cells, thus promoting cell lysis. C3a and C5a are powerful anaphylatoxins able to induce chemotaxis and inflammation.

In the serum and interstitial fluids, complement proteins circulate largely in an inactive form: however, in response to pathogen-associated molecular patterns (PAMPs) and/or damage-associated molecular patterns (DAMPs), they become activated through a sequential cascade of reactions (6) (7). The recognition of these highly conserved molecular patterns is achieved via different types of pattern recognition molecules (PRMs) (8) (Figure 1). The activation of complement system occurs via three different pathways: the classical pathway (CP), the alternative pathway (AP), and the lectin pathway (LP) (4). Independently from the signaling initiated, all the pathways lead to the formation of a central enzyme, the C3 convertase, that cleaves C3 into C3a and C3b. In the CP, immune complexes of immunoglobulin M (IgM) or hexameric IgG are recognized by C1q together with the associated proteases C1r and C1s (9). The LP contains six PRM: mannose-binding lectin (MBL), Ficolin-1, Ficolin-2, Ficolin-3, Collectin-10, and Collectin-11, which recognize carbohydrate and acetylated structures on pathogens and form a complex with MBL-associated serine proteases (MASPs) (10, 11). The AP is continuously activated at low level by the spontaneous hydrolysis of C3 called the “tick-over.” This mechanism generates C3b that can then covalently bind to various proteins, lipids, and carbohydrate structures on microbial surfaces (4). As examples of DAMP-mediated complement activation, we could mention the CP induction by C-reactive protein (CRP) or Pentraxin-3 (12) (8); in IgA nephropathy, LP can be triggered by IgA (13), and after ischemia/reperfusion injury (IRI), L-fucose induced LP on stressed cells (7). With regard to AP, the cleavage of C3 can be induced by neutrophil enzyme elastase or myeloperoxidase (MPO) (14). Progressive C3 activation results in the formation of the C5 convertase, which cleaves C5 into C5b and C5a. C5 is the initiator of the terminal step, and C5b merged together with the components C6 till C9 assembling the membrane attack complex (MAC) pores (Figure 1). In the last steps of complement activation, the MAC leads to the direct lysis of the pathogen or target cells. Interestingly, MAC can also trigger a range of non-lethal effects on cells as NLRP3 inflammasome activation in the cytosol (15). Complement activation also leads to the generation of other effector molecules such as opsonins (C4b, C4d, C3b, iC3b, C3dg, and C3d) and anaphylatoxins (C3a, C5a), which can interact with their respective receptors and recruits granulocytes, monocytes, and other inflammatory cells on site of infection (16). Anaphylatoxins can bind specific receptors expressed not only on PBMCs but also on parenchymal cells such as tubular epithelial cells within the kidney, initiating inflammation and chemotaxis (C3aR, C5aR1 and C5aR2) (17) (Figure 1).

However, complement functions have been implicated in the pathogenesis of disorders not necessarily related to infections such as cancer (18), neurodegenerative and age-related disorders [i.e., age-related macular degeneration (AMD)], metabolic diseases (2), the progression of chronic kidney disease (CKD) (19, 20), and more importantly renal aging (21, 22). Therefore, increasing efforts are necessary to evaluate the efficacy of targeting complement to arrest the progression of renal aging during CKD (20, 23).

## Local Production of Complement Factors at Renal Level

Complement factors are produced predominantly by the liver; however, some factors as C1q (24), properdin, and C7 (25) are released by leukocytes (26); in addition, adipocytes can synthesize factors B and D (also known as adipsin) (27). In the kidney, tubular epithelial cells can produce virtually all complement proteins (28). The percentage of tubular complement biosynthesis can increase significantly during inflammation (29–31). Following IRI, complement C3 can be expressed by proximal tubular epithelial cells (32), endothelial cells (33), glomerular epithelial and mesangial cells (34). The C3 messenger RNA (mRNA) upregulation and the subsequent biosynthesis has been demonstrated to play a central role in kidney transplantation (35, 36). Pratt et al. demonstrated that wild-type (WT) mice with intact serum complement activity do not reject allogenic C3-deficient kidneys, underlying that kidney-derived complement is a key mediator of renal injury (37, 38). Thus, complement can switch the immune system balance toward a persistent and proinflammatory response that, if directed against self-antigens, might promote the induction of autoimmunity or, if directed against donor antigens, might lead to rejection (23).

## Intracellular Complement Activation and EVs Carried Complement

Recent studies have revealed that complement activation is not confined in the serum or produced locally by resident and infiltrating cells into interstitial fluids. Complement cascade can also be initiated intracellularly. The intracellular complement activation, the Complosome, has been investigated mainly in human CD4 + T cells (2); however, it has also been described in adipocytes, monocytes, fibroblasts, B cell, and epithelial and endothelial cells. In resting T cells, the function of C3 and C5 intracellular activation has been associated to the homeostatic cell survival by keeping low level of mTOR signaling (2). Nevertheless, after T-cell receptor (TCR) activation, intracellularly cleaved C3 can induce the Th1 differentiation, the NLRP3 inflammasome activation, and the T cell metabolism reprogramming by regulation of glycolysis and mitochondrial oxidative phosphorylation (39). Interestingly, aging is also a process strongly integrated with chronic inflammation and metabolism; therefore, the recently discovered connection between the Complosome and cellular metabolome might add a new layer of complexity in the impact of complement intracellular activation in several aging-related diseases (as obesity) and in the acceleration of renal aging during CKD.

Lastly, complement components can be also identified in circulating extracellular vesicles (EVs), particularly in microvesicles (MVs) with a size ranging from 0.1 to 1 μM. EVs can carry and modulate complement system in several age-related disease, such as AMD (40), providing a new, extracellular way to deliver complement in different body compartments.

## Complement in Kidney Disease

The complement system is considered a crucial pathogenic mediator in the development of several renal diseases. The kidney is particularly susceptible to complement-mediated injury, mainly due to the ultrafiltration function, the low expression of complement regulators, and the local complement production (23). Complement aberrant activation, acquired or inherited dysregulation, and ineffective clearance have been observed in a wide spectrum of glomerulonephritis [lupus nephritis (41), C3 glomerulopathy, IgAN, antineutrophil cytoplasmic antibody (ANCA)-associated vasculitis], in thrombotic microangiopathy [atypical hemolytic uremic syndrome (aHUS)], in renal transplantation, and in the progression to CKD (20, 38). A predominant role for glomerular immunocomplex deposition has been observed in lupus nephritis with the involvement of CP, LP, and also AP. Moreover, the impairment of AP predominantly characterizes the aHUS and the C3 glomerulopathy. These findings have led to the clinical use of complement blocking therapeutics as Eculizumab in aHUS (42).

In the progression to CKD, the role of all the three pathways has been assessed, and promising results are coming from clinical trials. However, we are still far from the clinical use of complement inhibitors to delay the progression of renal fibrosis.

## AKI-To-CKD Transition: the Role of Complement

Acute kidney injury (AKI) characterized by a rapid loss of renal function and is still associated to a high morbidity and mortality (43). The most common causes of AKI include renal IRI, sepsis, or several exogenous nephrotoxins such as drugs. Currently, it is well known that AKI predisposes to the future development of CKD and subsequently to end-stage chronic renal disease (ESRD) (43). However, the cellular and molecular mechanisms underlying the progression from AKI to CKD remains incompletely understood.

Complement system was traditionally related to the early development of AKI (44); nonetheless, several evidence indicated that complement is a pivotal mediator of tubular senescence (21, 22) and interstitial fibrosis, the common hallmark of premature aging that characterizes the CKD (45). The major complement components involved in the AKI-to-CKD transition seems to be the anaphylatoxins C3a and C5a and the terminal C5b-9 that contribute to the damage during CKD progression through various mechanisms. After binding to C5aR and C3aR, these anaphylatoxins exert a proinflammatory and fibrogenic activity on tubular and endothelial cells (46, 47), pericytes (31, 48), and resident fibroblasts; moreover, they can mediate renal fibrosis by stimulating transforming growth factor beta 1 (TGF-β1) production in cultured murine tubular cells. As a consequence, activated endothelium, monocytes, and injured tubular epithelium (49) have all been shown to secrete profibrogenic factors such as TGF-β and platelet-derived growth factor (PDGF), able to activate resident fibroblasts promoting collagen deposition. In addition, we recently demonstrated that the complement anaphylatoxin C5a contribute to fibrosis inducing the pericytes to myofibroblast transdifferentiation (PMT) through pERK activation (48).

Other mechanisms of complement-mediated transition to CKD are the chemotactic effect on different infiltrating leukocytes (50) with the inhibition of the polarization of T-helper cells to Th1 cells (51) (52). The subsequent shift of T-helper cells to Th2 cells, together with their cytokines release, such as TGF-β, has been shown to act in a profibrotic manner (53). The predominant profibrotic effect of TGF-β signaling in AKI-to-CKD transition, in tubular cell cycle arrest, and myofibroblast transdifferentiation has been reviewed elsewhere (54).

Finally, the terminal C5b-9 complex is a powerful inducer of profibrotic and proinflammatory cytokines by a variety of renal cells. Incubation of human glomerular epithelial cells with sublytic doses of C5b-9 significantly increased the collagen synthesis (55) and the release of TGF-β1 and interleukin IL-6 (56). In addition, endothelial cells exposed to sublytic concentration of C5b-9 released profibrotic factors including fibroblast growth factor (FGF) and PDGF (57). Similar effects were observed in tubular epithelial cells; stimulating proximal tubular epithelial cells with C5b-9 led to increased expression of collagen type IV (58). Collectively, these *in vitro* evidence supported that C5b-9 can increase the profibrotic process associated with progressive renal injury. Uncontrolled complement activation may ultimately result in maladaptive tissue repair with irreversible development of fibrosis and renal aging.

## The Role of Complement in IRI

Recent improvements in immunosuppressive therapy have made kidney transplantation the treatment of choice for ESRD patients (59). Complement system might have a detrimental role in different phases of renal transplantation from brain (DBD)/cardiac death (DCD) in deceased donors, to organ procurement, to IRI, allograft rejection, until the chronic graft deterioration (60). Increased systemic levels of sC5b-9 were observed in DBD and DCD but not in living donors, which correlate with increased acute rejection in the recipients (61). Furthermore, a strong association between chronic graft injury and overexpression of complement components has been found by proteomic analysis in kidney donor biopsies (62). These results indicated that shorter periods of ischemia are clearly associated with less complement activation; in addition, the protein profiles of preservation solutions in which kidney from deceased donors had been stored revealed intense activity of complement effectors (as C3, factor B) during organ storage preceding transplantation (63).

Following organ procurement, the role of complement in renal IRI has been extensively investigated by several studies (64, 65). Importantly, renal IRI is the pivotal contributor in the development of delay graft function (DGF), traditionally defined as the requirement for dialysis during the first week after transplantation. IRI is initiated by the occlusion of blood flow that is necessary for organ collection and during hypothermic ischemia for the storage; in this conditions, renal cells are permanently damaged due to hypoxia, ATP depletion, and accumulation of metabolic waste, resulting in the production of reactive oxygen species (ROS) and DAMPs (i.e., histones, heat-shock proteins). Reperfusion leads to a more detrimental inflammatory response, resulting in further tissue damage characterized by early release of inflammatory cytokines such as IL-6, tumor necrosis factor alpha (TNFα), and IL-1α that represent a powerful inflammatory milieu capable to induce a cellular senescence-associated secretory phenotype (SASP).

**FIGURE 2:**
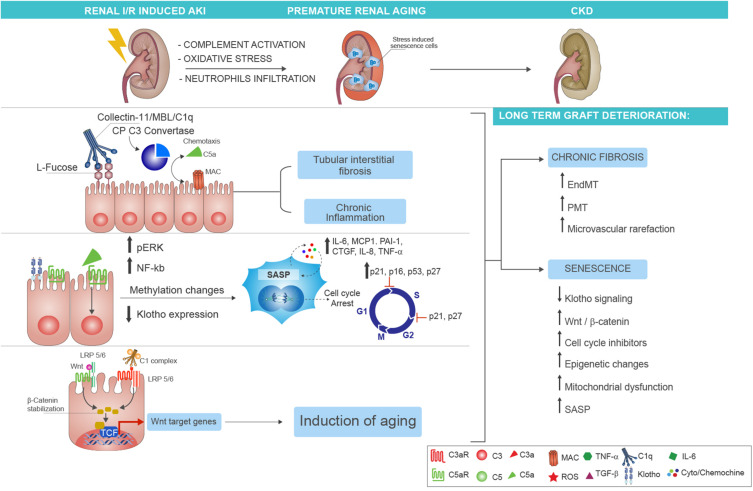
Complement-driven accelerated renal senescence after IRI-AKI leading to CKD progression. During renal ischemia/reperfusion injury (IRI), activation of complement may lead to reactive oxygen species (ROS) generation and neutrophils infiltration, thereby establishing a prosenescence microenvironment that promotes accelerated renal aging. Several molecular mechanisms can be responsible for the establishment of tubular senescence after complement activation. First, renal tubular epithelial cells expressed fucosylated glucose patterns upon IRI, which can be recognized by the lectin pathway pattern recognition receptor (PRR) (as Collectin-11), therefore inducing complement activation and tubular interstitial fibrosis with persistent chronic inflammation (upper part). Second, the release of C5a anaphylatoxin, through methylation changes, can induce cellular senescence characterized by growth arrest, inhibition of apoptosis, and acquirement of a senescence-associated secretory phenotype (SASP). The molecular mediators of the growth arrest are mainly G1-S cell cycle inhibitors as p16INK4a, p21 WAF/CIP1, p53, and p27. The p21 and p27 proteins can mediate the cell cycle arrest also during the transition from phase S to phase G2 of cellular cycle. The SASP is maintained and amplified by the increased expression of proinflammatory [such as interleukin (IL)-6, monocyte chemoattractant protein-1 (MCP-1), IL-8, PAI-1, tumor necrosis factor alpha (TNFα)] and profibrotic cytokines [as connective tissue growth factor (CTGF)] (in the middle). Finally, another molecular mechanism of complement-induced renal inflammaging is mediated by Wnt/β-catenin signaling. The C1 complex (that is composed by the association of C1q with C1s and C1r serine proteases), after binding the serpentine Frizzled receptor (indicated in red), can cleave the N-terminal domain of LRP5/6 and stabilize the β-catenin protein (in the left). The function to bind Frizzled receptors and to cleave the LPR5/6 extracellular domain is normally exerted by the Wnt protein (indicated in green). The β-catenin stabilization allows the nuclear translocation, the interaction with transcription factors, and the augmented gene expression of proaging Wnt target genes. The final and common event of all these complement-mediated pathways is represented by the generation of stress-induced senescence cells, indicated in the figure as enlarged cells, indicated as blue cells to highlight the positivity to SA-βGal enzymatic assay. The persistence of these cells and the higher increase in SASP chemokines result in chronic fibrosis by the induction of endothelial-to-mesenchymal transition (EndMT) and pericyte-to-myofibroblast transition (PMT) that can amplify microvascular rarefaction together with generation of new myofibroblast and proliferation of resident fibroblast. Lastly, several mechanisms are involved in the maintenance of renal inflammaging such as the downregulation of Klotho, the increased Wnt signaling, mitochondrial dysfunction, the epigenetic changes, and the increased and stable expression of cell cycle inhibitors.

A large body of evidence from both experimental (66–68) and clinical (20) studies has identified in complement activation a crucial mediator of chronic tubulointerstitial fibrosis following renal IRI (69). In the past years, using complement-deficient animals, the terminal C5b-9 was identified as principal inducer of tubular injury after IRI (70). In particular, Zhou et al. demonstrated that C3^–^-, C5^–^-, and C6^–^-deficient mice were protected against ischemic damage, whereas C4^–^-deficient mice were not (59). These initial findings underlined the importance of tubular (and not endothelial) injury in the I/R physiopathology. Next, we suggested a more significant role for the MAC and the AP pathway. The involvement of AP was also elegantly confirmed by Thruman et al. in transgenic mouse models (68, 71). More recent reports have focused on pattern recognition receptors of lectin pathway (LP-PRRs) (MBL, Collectin-11, Ficolin-3), CP-C1q, and C5aR1/C5aR2, indicating that all these complement components were able to trigger the IRI and fuel the progression to CKD (Figure 2). Hence, renal function in MBL-deficient mice was significantly preserved after IRI (67).

Furthermore, Collectin-11, a PRR that binds a ligand (L-fucose) (72) expressed on stressed tubular cells, was demonstrated capable to activate complement LP in C4-independent manner. This mechanism, called C2/C4 bypass, has been proposed by Yaseen et al. (73) and depends on the unique capacity of MASP2 to directly activate C3, leading to C3b and C3a fragment formation without the involvement of C4 or C2. These findings finally explained previous and contradictory results that showed protection from IRI in MASP2-deficient (74) but not in C4-deficient mice (70, 75, 76). More importantly, compared with wild-type, Collectin-11-deficient mice showed significantly reduced renal functional impairment and leukocyte infiltration, less chronic inflammation, and tubulointerstitial fibrosis after renal IRI (77). The analysis of other LP factors in patients showed that high pretransplant level of Ficolin-3 was strongly associated with poor allograft survival and age after kidney transplantation (78). In accordance, Ficolin-2 gene rs7851696 polymorphism influenced kidney allograft functions, with specific allele increasing the risk of DGF and rejection (79). These results revealed a central role of LP in the development of renal fibrosis after IRI, with strong clinical implication in the transition from AKI to CKD (77).

Even if the contribution of CP has been controversial (80), the CP and LP are effectively involved in development of early fibrosis. In a pig model of renal IRI injury, we demonstrated the deposition of C1q and MBL on peritubular capillaries colocalization with C4d after 15 min from reperfusion. The treatment with recombinant human C1-INH (C1 esterase inhibitor) (81), an inhibitor of CP and LP pathways, conferred protection not only reducing infiltrating cells but also modulating the generation of myofibroblasts by reducing endothelial-to-mesenchymal (EndMT) (82) and the pericytes-to-myofibroblast (PMT) transitions (48). Consistent with these results, Delpech et al. demonstrated that treatment by C1-INH appeared to be protective also after 3 months from IRI, reducing the development of chronic graft fibrosis (83). These data have been translated to humans, and the use of C1-INH in patients receiving deceased donor kidney transplants with high risk for DGF has been investigated in a recent clinical trial (84) and will be further discussed in the paragraphs below.

Next to C1 blockage, the C5aR1 and C5aR2 inhibition could offer promising results. C5aR receptors are expressed both on peripheral and infiltrating leukocytes (such as dendritic and T cells) and on renal parenchymal cells such as tubular epithelial cells, mediating the recruitment of leukocytes. Additionally, these receptors mediated allograft injury before (85) and after kidney transplantation (86) (Figure 2). Consistently, pathogenic roles for C5aR1 in renal tubulo-interstitial fibrosis have been reported in different models of renal IRI (66, 87–91), murine model of unilateral ureteral obstruction (UUO) (92), and chronic pyelonephritis (93). Remarkably, in clinical settings, donor urinary C5a concentrations before transplantation has been shown to be higher in the recipients with risk of DGF (92, 94).

Considering the C5aR1 profibrotic and complement-independent detrimental effect, the use of C5aR inhibitors should be taken into consideration. Recently, successful evidence are coming from trials using the Avacopan (CCX168), an orally administered, selective C5a receptor inhibitor in aHUS (NCT02464891), ANCA-associated vasculitis (NCT01363388, NCT02222155) (95), C3 glomerulonephitis (NCT03301467), and IgA nephropathy (13) (NCT02384317). The potential beneficial role of C5aR1 inhibition in the context of renal IRI should be encouraged.

## From Antibody-Mediated Rejection to CKD: the Role of Complement

After kidney transplantation, antibody-mediated rejection (ABMR) is one of the leading cause of long-term graft failure and CKD in which complement system plays a key role (96, 97). ABMR is characterized by glomerulitis, peritubular capillaritis, acute thrombotic microangiopathy tubular injury, C4d deposition in the peritubular capillaries, and microvascular inflammation (98, 99). As a consequence of these immunological attack to the graft, there is a significant increased incidence of late graft loss after ABMR (100–103). Even in presence of early acute rejection, several pathogenic mechanisms will progressively contribute to the later development of tubulo-interstitial fibrosis and progression to CKD (104, 105).

In the recipients, the presence of donor-specific antibodies (DSAs), i.e., natural IgM and IgG directed against donor endothelial human leukocyte antigen (HLA) or ABO antigens is referred as sensitization and represents the principal risk factor for ABMR. The immune complexes generated will activate CP by C1q-r-s complexes, therefore leading to covalent C4d deposition on peritubular capillaries. Consistently, for several years, the C4d deposition has been considered the gold standard for ABMR diagnosis; however, today, it is recognized that up to 55% of patients can develop ABMR without detectable capillary C4d deposits. Indeed, a C4d-negative ABMR phenotype has been included in Banff 2013 classification (106–109).

Interestingly, all complement pathways are involved in ABMR (110, 111), leading to recruitment of leukocytes such as natural killer cell, monocyte/macrophage-mediated damage, endothelial injury, and increased intragraft coagulation (112, 113). Besides HLA matching and alloimmune response, other factors can influence the development of ABMR as donor and patient ages, cardiovascular complications, time on dialysis, glomerular disease recurrence, or more commonly hypertension, dyslipidemia, proteinuria, anemia, and diabetes. Interestingly, a significant complement activation has been observed also in these conditions. Bobka et al. demonstrated an increased complement activation in pretransplant biopsies from diabetic, hypertensive, or smoking donors (97). The authors showed a predictive value of complement activation in donor biopsies for later outcome; effective analyses of these deposits in the donor were characterized by C1q, factor D, C3c, and C5b-9 and tubular MASP2 and Collectin-11 in kidney that would have developed ABMR. Interestingly, at the diagnosis of ABMR, the expressions of these complement component were associated with higher serum creatinine and morphological changes.

Although is not clear whether the complement deposition occurred already in the donor or during the following IRI, a role of intragraft complement release has been hypothesized. We do know from the animal model of acute renal transplant rejection that early complement deposition can be associated by local synthesis of complement. By performing renal transplantation of a donor C3^–/–^ kidney in a wild-type recipient mice (donor, C3^–/–^; recipient: wild type), Pratt et al. demonstrated a significant increased long-term survival and less rejection incidence compared to wild-type mice recipients transplanted with allogenic wild-type kidney (Wtype/Wtype). Therefore, locally synthesized C3 is the most important trigger of rejection than circulating C3 and a powerful inducer of chronic damage (92). Other experimental evidence to support the role of complement in rejection were provided by Wang et al. (114). In a mouse model of ABMR induced after heart transplantation, Wang et al. showed that C5 blocking prevented ABMR and allowed long-term renal function. In conclusion, all these data support the use of complement inhibitors as therapeutic strategy to prevent the long-term complications of ABMR.

## Complement and Renal Inflammaging: an Unexplored Field

### Complement in Aging Diseases

Complement activation has been investigated in diseases of aging such as Alzheimer’s and Parkinson’s disease, amyotrophic lateral sclerosis, and multiple sclerosis or AMD (115). For instance, polymorphisms in factor H are known to increase several folds the risk of AMD, the most common cause of irreversible blindness. In addition, C3 gene expression is upregulated with aging in humans (116). Furthermore, C1q levels, which mediate synapse elimination in CNS, are dramatically increased in aged brains (117).

Interestingly, systemic protein C1q level increases with aging and can activate the Wnt/β-catenin signaling that is primarily involved in mammalian skeletal muscle aging (118, 119). The canonical Wnt signaling is activated by two kinds of receptors: the Frizzled family of serpentine proteins and the single-transmembrane protein low-density lipoprotein receptor-related protein 5/6 (LRP5/6) (120, 121) (Figure 2). Recently, Naito et al. demonstrated that C1q-r-s complex, after binding to Frizzled receptors, could induce the N-terminal cleavage of the ectodomain of LRP6, thereby activating Wnt pathway (119). In renal tubular epithelial cells, we found that C5a induced aberrant methylation changes in Wnt signaling related genes and in particular in Frizzled 6 (*FZD6*) receptor gene (22). This unexpected role of complement C1q in inducing an impaired regenerative capacity of skeletal muscle in aged animals has been further confirmed by several studies (122) showing that C1q secretion led to muscle fibrosis (122) and induced an increased proliferation of vascular smooth muscle cells via β-catenin signaling. From these observations (123), a role of C1q in the development of arteriosclerosis and arterial stiffening that occurs in advancing aging has been hypothesized. By the analysis of the circulating C1q and other cytokines associated with cardiovascular diseases (as TNF-α and IL-6), there emerged a significant correlation between C1q and aging-induced arterial stiffness. Regarding the role of LP in aging, evidence from Tomaiuolo et al. (124) showed that the specific MBL2 gene haplotypes (in particular, the high-activity-associated haplotypes as HYPA and LYQA) were significantly lower in centenarians than in the general population. The investigators identified also a role of MBL in the clearance of senescent cells. However, the mechanism underlying this peculiar connection between reduced MBL levels and longevity deserves more investigations.

In the healthy subjects, the correlation between complement and aging has its roots in earlier studies (125). In 1978, Yonemasu (126) demonstrated that, in a cohort of healthy volunteers (from birth up to 75 years), C1q and C3 levels independently oscillated with age. C1q increased gradually from birth to 60 years, whereas C3 reached higher level at 1 year, decreased until puberty, and augmented steadily after this age. Accordingly, in another cohort, Nagaki et al. detected an increased levels of CH50 activity, C1q, and C3 and decrease in factor B in older healthy subjects (127).

From these studies emerged a predominant role of C1q-CP in physiological aging. However, more recently, the findings from Gaya da Costa et al. provided strong evidence that also the AP was significantly activated in the elderly (76). In addition, authors also revealed increased terminal pathway components with age: these results are in line with the capability of complement to contribute to the clearance of senescent cells by MAC deposition (128).

The link between complement activation and physiological aging has been clarified in several experimental knockout models. Qiaoqiao Shi et al. (129) demonstrate that C3-deficient mice were protected from the synapse, neuron loss, and cognitive decline typically observed in older mice, suggesting an important role of C3 in the aging brain. Accordingly, in a model of AMD, CD59a^–/–^ mice showed an age-dependent increased expression of activators of the alternative complement pathway (C3, FB, FB) in the retinal pigment epithelium (RPE) choroid (130).

Furthermore, an age-related increase in complement C1q, C4, C3, and factor B expression was found in wild-type mouse brain (116).

All together, these studies demonstrate that aging is linked to a dysregulation of complement system, in particular of CP and AP, therefore to a progressive impairment of immune response.

Moreover, aging is associated to the establishment of a proinflammatory milieu generated by the hypersecretion of several cytokines [TNFα, IL-6, monocyte chemoattractant protein-1 (MCP-1), PAI-1] associated to higher risk for cardiovascular morbidity and mortality (131, 132).

More importantly, premature renal aging immediately after kidney transplantation could be modulated by soluble and circulating factors and, virtually, also by complement system. Liu et al. (133) showed that blood from young mouse was able to reduce IRI-induced AKI in older mouse (134). Using an experimental model of parabiosis, a surgical procedure that allowed a shared circulation between older and younger mice, Liu et al. demonstrated that a youthful systemic milieu was able to attenuate inflammation, oxidative stress, and apoptosis after renal IRI (133). These results are in line with previous findings demonstrating that bone marrow from young donor mice alleviated renal aging (135) and with recent data indicating that transplantation of young bone marrow can rejuvenate the hematopoietic system and preserved cognitive function in old recipient mice (136, 137).

### Mechanisms of Renal Inflammaging

The term *renal senescence* reflects the complex interplay between genetics, immunological, and hormonal factors able to lead to structural and functional changes observed in aged kidneys (138).

During physiological aging that occurs in the elderly, a low-grade of systemic inflammation and the dysregulation of innate and acquired immune responses are normally observed. This systemic, chronic proinflammatory status has been defined for the first time by Claudio Franceschi as inflammaging, and the associated immunological impairment has been named immunosenescence. [all reviewed in more detail by Franceschi et al. (139)]. Inflammaging is a risk factor for multiple chronic diseases, such as CKD, cardiovascular diseases, cancer, depression, dementia, osteoporosis, sarcopenia, and anemia. Besides physiological aging, several mechanisms can induce inflammaging such as oxidative stress, mitochondrial dysfunction, complement activation, DNA damage, changes to microbiota composition, NLRP3 inflammasome activation, visceral obesity, and cellular senescence. In the kidney, inflammaging has been strongly connected to tubular senescence, characterized by cell cycle arrest and the acquirement of a SASP. The common features of renal aging have been observed in a wide range of kidney disorders as pretransplant cold storage preservation, IRI, ABMR, diabetic nephropathy, and IgA nephropathy (114). Histological features of kidney aging include glomerulosclerosis, interstitial fibrosis, glomerular basement membrane thickness, microvascular rarefaction, and tubular atrophy. Interestingly, similar changes are also observed in transplant injured kidney, suggesting that maladaptive repair after acute insults can be considered as the fuel for kidney inflammaging (140).

The SASP cell secretome involves the increased release of a large spectrum of proinflammatory [IL-6, IL-1α, IL-1β, IL-8, MCP-1, C–X–C motif chemokine ligand 1 (CXCL-1)], profibrotic [TGF-β, connective tissue growth factor (CTGF)] cytokines, growth factors (fibroblast growth factor 2 and hepatocyte growth factor), and matrix metalloproteinases (MMPs) (141). These factors acting on neighboring health cells and in the circulation exacerbate the progression of the inflammation, lately of the fibrosis and then progression to CKD (138, 142) (Figure 2). Healthy aging must rely on the ability to maintain a balanced immunological response between pro- and anti-inflammatory factors, allowing the inflammation resolution in a timely effective manner (143). In senescent cells, the persistent, chronic inflammaging is maintained by controlled downregulation or unchanged stable levels of anti-inflammatory cytokines as IL-10, IL-4, IL-2, IL-11, IL-12 or Fractalkine (CX3CL-1). For that reason, another well-described consequence of the SASP secretome is the tumor initiation and progression in cells residing in proximity of senescent cells (141). The list of molecular processes involved in premature kidney aging is complex; below, we will focus on the main processes that have been shown to link inflammaging with renal transplantation and complement system such as Klotho signaling, Wnt/β-catenin pathway, increased expression of cell cycle inhibitors, epigenetic changes, and mitochondrial dysfunction (144).

### Klotho and the Aging Kidney

The Klotho protein, expressed predominantly in epithelial distal convolute (DCT) and proximal tubules, is an antisenescence factor. Although the transmembrane form of Klotho functions as a coreceptor for FGF23 signaling, the extracellular domain is cleaved and released into the blood, urine, and the cerebrospinal fluid acting as an endocrine factor on several distant organs, such as the heart (145).

Klotho gene is strongly involved in human aging and longevity. For instance, Klotho-deficient mice exhibit a shortened life span, skin and muscle atrophy, cognitive impairment, osteoporosis, and hearing loss, resembling an accelerated aging phenotype (146). In contrast, overexpression in Klotho gene in transgenic mice has been associated to increased life span (147, 148). In human, serum levels of Klotho decrease with age and are downregulated in several forms of AKI and chronic kidney injury (149–153). The principle function of Klotho, which acts in the FGF 23 signaling, is mainly implicated with calcium, phosphate, and Vitamin D metabolism, explaining the central involvement in aging-related-vascular calcification and osteoporosis (154).

A huge body of literature describes the reduced Klotho expression in the kidney, blood, and urine after IRI in mouse (155, 156), rat (155, 157, 158), and swine (21) models. Hu et al. (155) induced IRI in mice with different genetic background that led to various endogenous Klotho levels ranged from heterozygous Klotho haploinsufficient (with low/absent Klotho expression), to wild-type (WT, normal Klotho expression), to transgenic mice overexpressing Klotho. Compared with WT mice, after I/R, Klotho levels were lower in haploinsufficient and higher in transgenic. In addition, the haploinsufficient mice had more deleterious functional and histological damage compared with WT mice, whereas these changes were milder in overexpressing transgenic mice. These results support the concept that reduced Klotho levels predispose the kidney to injury, accelerating renal fibrosis, and senescence, therefore promoting to transition from AKI to CKD (159).

In accordance, the restoring of Klotho level by exogeneous supplementation has been demonstrated to be renoprotective from fibrosis, senescence, and apoptosis (157). Although the Klotho expression was spontaneously restored with recovery in the WT [after 7 days from IRI (155)], preventing the early Klotho drop is crucial to avoid or to delay the AKI-to-CKD progression, together with cardiovascular complications (156). Different methods of Klotho supplementation have been evaluated, from exogenous administration of recombinant α-Klotho (155, 156) to forced expression by adenoviral vectors (157, 160), to minicircle vectors that allowed self-production of Klotho protein in the cells (161).

Other therapeutic strategies to reduce the Klotho loss with significant limitation of chronic damage could arise from complement inhibition. Our group recently demonstrated in a pig model of IRI significant downregulation of Klotho by 24 h from injury; importantly, Klotho was efficiently preserved after treatment with C1-INH, which efficiently modulated nuclear factor kappa B (NF-kB) signaling (21). Furthermore, the C5a anaphylatoxin led to a significant Klotho protein and gene expression decrease through a mechanism mediated by NF-kB (21). In addition, tubular cells exposed to C5a acquired a senescent phenotype as demonstrated by increased SA-βgal positivity, cell cycle arrest induced by increased p53, p21, and p16, and the acquirement of a SASP as detected by *IL-6, MCP-1*, *CTGF*, SERPINE 1 (*PAI-1*) gene expression. Interestingly, C5aR1 inhibition by monoclonal antibody protected the tubular cells from senescence (22).

Between all the cytokine involved in the SASP development, PAI-1 is also an essential mediator of cellular senescence (162) and could offer a target to counteract renal inflammaging. PAI-1 is expressed in senescent cells and tissue and is particularly highly increased in Klotho-deficient (kl/kl) mice. Furthermore, PAI-1 can be induced by C5a in human macrophages (163) and renal tubular cells (22). Using Klotho- and PAI-1 deficient mice (kl/kl^–/–^pai-1^–/–^) (164), it was demonstrated that PAI-1 deficiency in kl/kl^–/–^ led to reduced senescence, preserved organ structure, and function with a fourfold increase in lifespan. Therefore, PAI-1 could be considered as a downstream effector of the IRI-induced Klotho loss; both the PAI-1 inhibition, by the development of selective PAI-1 antagonists (such as TM5441), together with the C5a blocking, could offer a new possibility to modulate the impairment in Klotho expression (165).

### Wnt/β-Catenin Pathway in Renal Aging

Wnt/β-catenin signaling, a pathway involved in organ development, normally is kept silent in normal adult kidneys (166) but reactivated during aging (118), renal tubulointerstitial fibrosis (167), vascular calcification, and progression to CKD (121, 168, 169). Wnt signaling is antagonized by the protein Klotho that can bind to multiple Wnt ligands and inhibit the signal transduction mediated by Frizzled receptors (118, 170).

Recently, Luo et al. (171) identified a predominant role for component Wnt9 in promoting renal fibrosis by accelerating tubular senescence both in human and in experimental model of renal IRI and CKD (171). Interestingly, Wnt9a expression level correlated with the extent of tubular senescence and interstitial fibrosis and, functionally, with decline of estimated glomerular filtration rate (eGFR). The Wnt/β-catenin signaling constitutive activation has already been demonstrated to induce myofibroblast activation in the absence of other type of injury (172), with Wnt4 playing a pivotal role in chronic fibrosis (173). We have already discussed the capacity of C1q to activate Wnt signaling, leading to mammalian aging. Our *in vivo* studies confirmed that renal IRI activated Wnt4/β-catenin signaling, whereas the C1-INH treatment, blocking CP and LP, abrogated Wnt4/β-catenin activation preventing renal senescence and inflammaging (22). Lastly, in renal tubular cells, mitochondria are essential for energy production and are dysfunctional in AKI and CKD, leading to fibrosis and accelerated aging. Recent evidence indicated that Wnt/β-catenin signaling mediates age-related renal fibrosis and is associated with mitochondrial dysfunction (174) (Figure 2).

### Cell Cycle Arrest and Renal Senescence

Tubular epithelial cells have a great regenerative potential after an ischemic or toxic injury (175) (176). Early after an episode of AKI, in damaged tubular cells, cell cycle is arrested by specific inhibitors in order to provide time for DNA repair, avoiding exaggerate progression to apoptosis. However, after IRI induced AKI, the prolonged injury can lead to a permanently arrested cell cycle maintained by a persistent increase in cell cycle inhibitors. Cell cycle arrest is a common marker of cellular senescence and is regulated by three major proteins belonging to cyclin-dependent kinase (CDK) inhibitors: p16^*ink*4*a*^, p21^*waf*1/cip^, and p53 (177) (Figure 2). p16^*ink*4*a*^, encoded by the Ink4a/Arf locus, also known as CDKN2A, binds the kinases CDK4 and CDK6 that are necessary for cyclin D activation, therefore arresting cell cycle in G1 phase (178); the pivotal role of p16^*ink*4*a*^ in multiorgan aging has been revealed by Baker et al. (179). Interestingly, the elimination of naturally occurring p16^*ink*4*a*^-positive cells during physiological aging attenuated glomerulosclerosis and tubular senescence, extending lifespan. In rodents models of renal I/R, several evidence have been provided for p16 ^*ink*4*a*^ involvement in long-term graft deterioration (180–182). In particular, Braun et al. (180) demonstrated that after IRI, p16ink4a-deficient mice showed less interstitial fibrosis and tubular atrophy. Furthermore, p16^*ink*4*a*^(-/-) mice were associated with improved renal function, preservations of nephron mass, and transplant survival compared with wild-type controls. Consistently, mice that received kidney transplants from p16^*Ink*4*a*^ (-/-) donors had significantly better survival and developed a reduced amount of tubulointerstitial fibrosis (180). Similar results were obtained by other groups (182) even if some discrepancies exists in term of timing of p16 increased expression (181) or in correlation to the type of injury (183).

These results, describing the crucial role of p16 in mice model of aging, were confirmed in human kidney biopsies. In a seminal paper, Melk et al. (184) provided evidences that in normal human renal biopsies, nuclear p16INK4a staining was increased with aging. However, transplanted kidney with interstitial fibrosis and tubular atrophy or transplanted biopsies with chronic allograft dysfunction, exhibited a strongest nuclear and cytoplasmic staining, beyond the level expected from physiological aging. From this initial study, the hypothesis that the assessment of senescence by p16 measurement in time zero kidney biopsies could have a value for the prediction of chronic renal dysfunction in the recipient was investigated by other groups (185–187).

Another cell cycle inhibitor is p21^*WAF*1/Cip1^, a protein that after binding to CDK2, can block the CDK2-cyclin E complex, therefore arresting cell cycle in G1/S checkpoint. Megyesi et al. (188) demonstrated the role of p21 in tubular interstitial fibrosis and CKD progression in proximal tubular cells. In large experimental models, using an *ex vivo* hemoperfusion of pig kidneys after I/R, cold preservation, and machine perfusion, Chktoua et al. (189) found an increased p16 and p21 expression at tubular level after 180 min of reperfusion. In contrast with these results, in our swine model of renal I/R, p16 increased expression was not detectable before 24 h from reperfusion, and interestingly, the p16 and p21 protein level appeared to be modulated by C1-INH treatment (22). In accordance with these findings, C5a stimulated renal proximal tubular cells and exhibited a higher increase in p21 protein after both short time (3 h) and longer time (24 h) of C5a exposure. However, p21 seemed to be downregulated after 24 h of C5a exposition, followed by 24 h of normal culture, indicating a potential recovery of tubular cells. These *in vitro* results are in line with findings that indicated that p21 could transiently increase after injury (190), describing that p21 is essential for the beneficial effects of renal ischemic preconditioning. Temporary cell cycle arrest induced by a p21-dependent pathway could be important for subsequent tubular cell proliferation after I/R (190, 191). To confirm the establishment of cellular senescence, we also assessed the p16INK4a protein level. Stimulation with C5a significantly induced a constant augment in protein expression of p16INK4a compared to untreated condition (22).

### Complement and Epigenetic Changes in Aging

Epigenetic modifications are stable, heritable, and reversible genome changes that occur without the presence of alterations in the original DNA sequence (192). These modifications include DNA methylation, histone, phosphorylation, acetylation, methylation ubiquitylation, sumoylation, and miRNA pattern variations (193). There is an emerging evidence that epigenetics is crucial in healthy and accelerated renal aging (194). Not only physiological environmental factors (i.e., diet, exercise, education, and lifestyle factors) (195) but also acute inflammation, oxidative stress, or uremic toxins can contribute to susceptibility to CKD progression by epigenome changes (196, 197).

During transplantation, several stressors such as IRI, cold ischemia, and acute rejection can induce aberrant DNA methylation changes with serious implications for graft outcomes and acceleration of renal aging (198) (Figure 2). A great body of evidence recently provided the epigenomic, transcriptomic, and proteomic signature that characterize the biological older allografts (199–202) and the CKD methylation patterns (203, 204). Comparable results showing the importance of epigenetic modifications in AKI-to-CKD progression were obtained by rat and mice model of IRI, CKD, and premature renal aging.

Shasha Yin et al., in a mouse model of UUO, demonstrated that TGF-β can inhibit Klotho expression by epigenetic mechanisms leading to progression to renal fibrosis; TGF-β induces aberrant expression of DNMT1 and DNMT3a through inhibiting miR-152 and miR-30a, subsequently leading to Klotho promoter hypermethylation and Klotho protein suppression (205). In a rat model of IRI, Pratt et al. (206) found aberrant methylation in the C3 promoter gene in response to 24 h of cold ischemia and a subsequent 2 h of reperfusion, indicating an increased C3 release, therefore an amplification of local complement activation following the oxidative stress. However, these studies neither demonstrate a correlation between C3 aberrant methylation and increased gene expression (207) nor provided clinical translation data (208).

Recently, Denisenko et al. (209), in rat old kidneys, found an abnormal epigenetic pattern of extracellular matrix laminins that are involved in the development of glomerulosclerosis and tubulointerstitial fibrosis. *In vitro*, a predominant role for DNA methylation changes was identified by Bechtel et al. (210), who correlated the hypermethylation of *RASAL1*, a gene encoding an inhibitor of the RAS oncoprotein, with the fibrogenesis in the kidney. In our studies, we demonstrated that complement component C5a can induce a global tubular epithelial cell DNA hypomethylation (22), as observed in premature and accelerated renal aging (195, 211, 212). Furthermore, we found that C5a induced methylation modification-regulated genes involved in the prosenescence Wnt/β-catenin pathway and induced a SASP phenotype and cell cycle arrest (22) (Figure 2).

## Cell-Specific Effects of Complement in AKI-To-CKD Transition

### Renal Tubular Epithelial Cells and Complement

The impairment of tubular function is considered a critical step in many cases of AKI (213). During tubular injury, tubular cells dedifferentiate to replace the lost epithelial cells, but some of them fail in the recovery process and continue to produce factors that stimulate inflammation leading to fibrosis. This maladaptive response contributes to the development of CKD (214).

Activation of complement factors on tubular epithelium (215) is considered a key factor in tubulointerstitial inflammation and in the progression of renal dysfunction (216). Proteinuria is a common feature of kidney transplantation, and the association between proteinuria, complement activation, and tubulointerstitial fibrosis is well established (217). Indeed, the proteinuric condition provides a source of complement proteins to renal tubuli with amplification of the cascade (20).

**FIGURE 3:**
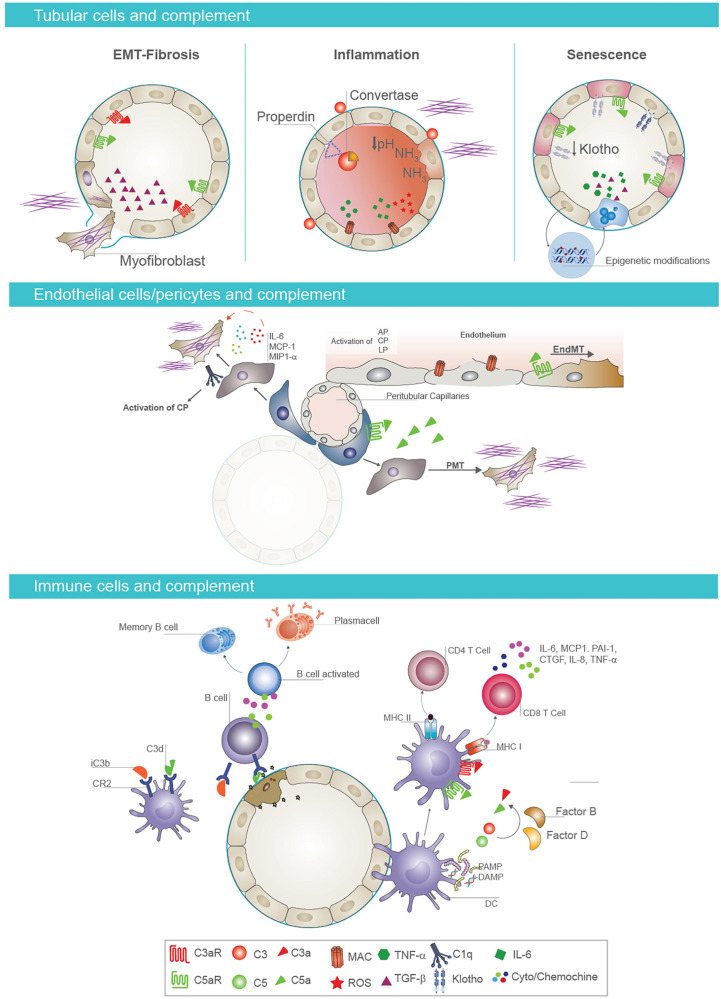
Cell-specific effects of complement in AKI-to-CKD transition. Tubular epithelial cells and complement activation (first panel). Activation of complement mediators on tubular epithelium is considered a key factor in renal fibrosis, inflammation, and senescence. Proximal tubular epithelial cells synthesize most components of the activation cascade as C4, C2, C3, factor B, and factor H. Reperfusion of the kidney following ischemia induces endothelial activation and release of nitrous oxide, leading to vasodilatation and leakage of complement components into the interstitial space In addition, complement proteins can be abnormally filtered across the altered glomerular barrier, leading to intratubular deposition of C3 and formation of membrane attack complex (MAC). When tubular cells are exposed to C3a and/or C5a, they synthesize transforming growth factor beta (TGF-β) that consequently promotes the EMT and fibrotic processes (on the left). Properdin, a key regulator of the complement system, enhances alternative pathway activation on the apical surface of tubular epithelium. Urinary pH and ammonia released from stressed tubular cells directly activate complement factor C3. This local complement activation and subsequent deposition of MAC on tubular cells induces a significant production of proinflammatory cytokines, contributing to renal inflammation (in the middle). Complement activation also induces a decrease in tubular expression of Klotho protein, an important antiaging factor. Complement promotes the acquirement of senescent tubular phenotype through epigenetic mechanisms, as DNA methylation (on the right). Endothelial cell/pericytes axis and complement activation (second panel). Complement also primes fibrotic process by inducing endothelial-to-mesenchymal transition (EndMT) and pericyte-to-mesenchymal transition (PMT) processes. In particular, C5a enhances EndMT process, causing phenotypic changes, with a decrease in endothelial markers and gain of fibroblast markers. In addition, pericytes, after C5a stimulation, acquire myofibroblast phenotype contributing to kidney fibrosis. Immune cells and complement (third panel). Complement components influence immune response in renal parenchyma. The binding of C3 fragments, iC3b and C3dg, to CR2 on B cells modulates B-cell response, increasing their activation and the development of memory B cells. Follicular DC also expressed CR2 and bind C3 fragments. After renal injury, PAMP and DAMP induce an increased expression of C3aR, C5aR1, and MHC class II on the surface of follicular DC and the synthesis and secretion of complement components C3 and C5 and factors B and D with local generation of C3a and C5a. These anaphylatoxins are strongly required for T-cell stimulation and activation in renal parenchyma.

The increase in albumin, which is associated to a higher risk of adverse transplant outcomes (218), compromised the balance between complement activation and inhibition, reducing factor H binding at tubular level (219). Several data also showed that urinary pH or ammonia released from stressed epithelial cells directly activated C3 (20) (Figure 3).

Recent studies indicated a key role of properdin in complement activation and in progression of proteinuria-induced tubulointerstitial injury. Properdin binds the glycosaminoglycans of the apical surface of tubular epithelium and stabilizes the AP convertase, enhancing AP activation. Then, interfering with properdin binding to tubular cells may provide a therapeutic option for the treatment of renal disease and prevention of CKD progression (20) (Figure 3).

Complement-cleavage products, C5a and C3a, are important mediators of renal inflammation and injury (20). These mediators bind their receptors C3aR and C5aR expressed on renal tubular, endothelial, and innate immune cells. When tubular cells were exposed to C3a and/or C5a, they synthesized collagen I and acquired a mesenchymal profibrotic phenotype contributing to renal fibrosis (47). The effects of C5a and C3a on tubular cells were mediated by TGF-β synthesis that consequently promoted epithelial-mesenchymal transition (EMT) (220). In accordance, studies in rodent knockout showed that the absence of C3aR and C5aR on renal tubular epithelial cells or circulating leukocytes attenuated renal IRI. Treatment *in vivo* using antagonist for C3aR and C5aR and for factor B could improve graft survival, reducing the decrease in renal injury, tubular apoptosis, and inflammation (216, 220, 221).

In addition, there are evidence that complement might contribute to renal injury in diabetic nephropathy. Complement activation and subsequent deposition of MAC on tubular epithelial cells induced a significant production of proinflammatory cytokines, as IL-6 and TNF-α, ROS, and components of matrix that contributed to amplify renal injury and fibrosis process. In this setting, tubular cells increased the expression of histocompatibility antigens stimulating T-cell response and autoimmunity process (20).

Therefore, complement has to be considered one of the principal actor in the progression from AKI to CKD, and its modulation could prevent tubular dysfunction. In our previous studies, we demonstrated the pathogenic role of the complement cascade in a swine model of IRI (222). We showed the link between oxidative stress/NOX activity, complement activation, and EMT process at tubular level (223). We also demonstrated the ability of C1-INH to reduce tubular dysfunction with prevention of I/R-induced renal injury (82) (Figure 3).

### Endothelial Cells and Complement System

Several studies highlighted the interactions between complement and the endothelium in pathogenesis of different renal diseases, including IRI, hemolytic uremic syndrome, and renal allograft injury (224). During inflammation, endothelium is continuously exposed to autologous complement (225) generated by the local or systemic activation of all complement pathways. Complement components such as C1q, C3a, C5a, and C5b-9 have direct effects on endothelial cells impairing their function. It is well known that C5b-9 not only induces cell lysis but also stimulates endothelial cells to acquire a prothrombotic cell surface. Furthermore, C5b-9 also contributes to platelet clumping as well as increased leukocyte adhesion and subsequent proinflammatory cytokine release (226) (Figure 3).

Accordingly, our group demonstrated that complement was primarily activated on peritubular and glomerular capillaries in a swine model of renal IRI, suggesting that endothelial cells are the primary target of injury (222). We also investigated an intriguing pathogenic process named EndMT in a swine model of renal IRI (82). EndMT has been shown to play a significant role in cardiac fibrosis, in arteriovenous fistula stenosis (227), and also in the recruitment of carcinoma-associated fibroblasts (228–230). In this model, a relevant portion of activated fibroblasts coexpress the endothelial marker CD31, indicating that these fibroblasts likely carry an endothelial imprint. This observation was also supported in renal diseases such as diabetic nephropathy (231–233) by colabeling the tissue with the endothelial marker CD31 and the fibroblast markers α-smooth muscle actin (α-SMA) and fibroblast-specific protein 1 (FSP1). In our study, we found that complement played a central role in this pathogenic process regulating fibrosis development within the graft (82). We also showed the effects of C1-INH in preventing C5b-9 deposition along peritubular capillaries, decreasing endothelial dysfunction and subsequent fibrosis (47, 222) (Figure 3).

A number of recent studies have shown that diseases of the vasculature and kidneys, including CKD, are associated with increased numbers of circulating endothelial microparticles and complement activation (224). EVs are actively shed from cells in response to injury. In particular, the microparticles found in the plasma of CKD patients presented increased levels of factor D that contributes to alternative pathway activation and systemic inflammation. Interfering with complement activation and microparticle release may be a potential therapeutic strategy to ameliorate kidney dysfunction in these patients (224).

In aHUS, complement-mediated injury is particularly active in renal glomerular capillaries and arterioles (234). Circulating complement fragments and local renal complement production lead to uncontrolled complement activation that induced platelet, leukocyte, and endothelial cell activation and systemic thrombotic microangiopathy with end organ damage or failure (235).

### Pericytes Dysfunction Upon Complement Activation

The tubular interstitial fibrosis and glomerulosclerosis are considered the principal responsible for progression of renal disease. The principal source of interstitial fibrosis in kidney disease is represented by activated fibroblasts, named myofibroblasts (236). These cells derive from different precursors such as renal resident fibroblasts, endothelial cells, tubular cells, circulating bone-marrow-derived cells and pericytes (237). Recent studies highlighted the role of pericytes in the pathogenesis of renal fibrosis (238). Numerous secreted factors are involved in the generation and persistence of fibrotic process such as TGF-β, VEGF, CTGF, MMP, WNT ligands, and PDGF (31) (Figure 3).

Recent advances demonstrated that complement system not only contributed to local renal inflammation and adaptive immune response but also primed fibrotic process (239). Specifically, Xavier et al. demonstrated a local synthesis and secretion of C1q, C1r, and C1s by PDGFRβ-positive pericytes in two different animal model of CKD (31). Moreover, they showed that the C1q released by UUO-mice pericytes was associated to increased expression of extracellular matrix components, collagens, and augmented Wnt/β-catenin signaling, all common hallmark of myofibroblast activation. Finally, the C1q local synthesis amplified interstitial inflammation by the release of IL-6, MCP-1, and macrophage inflammatory protein 1-alpha (MIP1-α) that in turn contributed to fibrosis by macrophages recruitment (31, 240).

In addition to C1q, we recently demonstrated for the first time that also complement component C5a promoted the PMT, amplifying tubulo-interstitial fibrosis (48). *In vitro*, C5a-exposed pericytes downregulated the constitutive marker PDGFR-β and upregulated aSMA^+^ stress fibers, the collagen I production, and the CTGF expression by TGF-β signaling. The C5aR blocking counteracted the PMT, reduced the C5a-induced collagen production, and more importantly inhibited the TGF-β pathway. In a swine model of I/R injury, we observed that C1-INH, acting upstream of C5 activation, indirectly reduced the release of C5a, preventing PMT process and ameliorating progressive kidney disease (48). Furthermore, also C5aR1^–/–^ were spared from PMT in a mouse model of bilateral I/R.

These data indicate that pericytes are an important source of complement components at renal level and expressed receptors for complement anaphylatoxins. Therefore, pericytes are pivotal target for complement inhibition therapy to delay progression from AKI to CKD.

### Immune Cells and Complement System

Next to direct effects on renal resident cells, complement components can influence the priming of alloantigen-specific immunity, modulating the interaction between dendritic cells (DCs) and T lymphocytes.

DCs are able to initiate an immune response by stimulating naive T cells, regulating the balance between Th1 and Th2 responses (241, 242). Moreover, complement components cooperate with DC to modulate T-cell response, and DCs themselves express complement factors, receptors, and regulators (243). Accordingly, we have demonstrated that C1q impaired DC activation leading to a limited T-cell response and preventing the overall immune response (244) (Figure 3).

Since the renal microenvironment has a strong influence on DC behavior, recent studies demonstrated the impact of local complement C3 on the differentiation and activation of DC. DC are considered the principal constituent of the tubulointerstitial compartment (245), and they produced C1q (41) and C3 in quantities similar to macrophages (246–248). Therefore, the contribution of C3 produced by these APC is strongly required for T-cell stimulation and activation in renal parenchyma. As a consequence, in C3-knockout organ, DCs have a reduced surface expression of major histocompatibility complex (MHC) class II and CD86, and they produce less IL-12 leading to a decrease in T-cell responsiveness (240, 249). Then, T-cell stimulation was reduced, and there was a shift for the generation of regulatory T cells (Figure 3).

This observation was confirmed *in vivo* in a skin allograft model. In this setting, infusion of mice with C3-knockout DC resulted in a less vigorous rejection of the skin allograft compared to mice infused with wild-type DC (248). Production of C3 has also been demonstrated for human monocyte-derived DC (240). The development of human monocyte-derived DC in either normal or C3-deficient human serum resulted in a reduced expression of HLA-DR, CD1a, CD80, and CD86 in the absence of C3, leading to a reduced responsiveness upon lipopolysaccharide (LPS) activation (240).

Finally, other studies clearly demonstrated that generation of C3a and signaling through C3a receptors was a very important event at the interface between DC and T lymphocytes interaction and had a major role in immune activation (240, 249).

Moreover, it is well known that complement receptor type 2 (CR2, also known as CD21) is expressed on B cells and follicular DC, and it binds C3 fragments iC3b and C3d,g when associated to antigens (250). This binding modulates B-cell response, and the blockade of CR2 may be a potential strategy to reduce immune response in renal transplantation. Interestingly, the increase in C3 plasma levels also controls the development of memory B cells in kidney diseases (251).

Follicular DC can arise anywhere in the body, during chronic inflammatory reactions. Krautler et al. showed in a murine model of chronic inflammation that mature follicular DC localized in renal tissue and generated from tissue intrinsic precursors (252). Therefore, this finding proved that follicular DC may be ubiquitous and could regulate renal local immune response. These observations point toward an important role of complement activation at the immunological synapse and the contribution of complement regulators in this process.

Notably, C3aR and C5aR1 are extra- and intracellularly expressed in human CD4 + T cells and regulate the activation of mTOR pathway and NLRP3 inflammasome (109). In a mouse model of renal transplantation, the expression of these two receptors have been reported in regulatory T (Treg) cells, and they are shown to drive Th1 cells maturation and activation.

Moreover, PAMP or DAMP induced an increased expression of C3aR, C5aR1, and MHC class II on the surface of DC (109, 253), and the synthesis and secretion of complement components C3, C5, and factors B and D can locally generate C3a and C5a. Several data showed that both C3a and C5a stimulated CD4 + T cells to release interferon gamma (IFNγ) and IL-2 and induced TH1 and TH17 cell responses. Moreover, CD4 + T cells secreted IFNγ also upon C5aR1 activation (253). Therefore, therapeutic blockade of either C3aR or C5aR1 signaling could induce human tolerance to alloantigens and may prolong allogenic graft survival. Altogether, these data strongly suggest that the inhibition of complement acting on immune cells may represent a potential target for preventing rejection and progression of kidney diseases.

## Complement Targets Strategies in Kidney Transplantation to Prevent AKI and Progression to Chronic Disfunction

The involvement of complement in a broad range of disease processes renders this system an interesting and promising target for therapeutic interventions (254). Several clinical trials evaluating dozen of candidate drugs targeting specific complement’s components are ongoing to date; most of them act as protein-protein interaction inhibitors, while others are physiological regulators or act on the genetic level, impairing the production of complement components (42). Eculizumab, the humanized monoclonal IgG2/4-antibody targeting C5, was the first complement drug available in the clinic, approved by the Food and Drug Administration (FDA) for the treatment of paroxysmal nocturnal hemoglobinuria (PNH) in 2007. PNH is a life-threatening disease characterized by an intravascular hemolytic anemia due to the destruction of red blood cells mediated by the complement system. In 2011, Eculizumab was also approved in the treatment of aHUS, where an uncontrolled activation of AP of the complement system (mutations in the complement regulatory proteins or acquired neutralizing autoantibodies against these regulatory factors) leads to a systemic thrombotic microangiopathy (255).

More than 10 years from its approval, the off-label usage of Eculizumab has been impressive, and several clinical trials are still assessing the potential indications, such as in kidney transplantation (42). As previously described, complement plays a major role in the IRI, such as DGF after kidney transplantation, and may be involved in the maladaptive repair leading to the progression to renal fibrosis and CKD (256). The role of Eculizumab in preventing and treating aHUS recurrence (255, 257) or *de novo* aHUS after kidney transplantation is well established to date (258). Recent evidence suggested its efficacy in the treatment of severe, progressive ABMR, or preventing ABMR in recipients with positive crossmatch against their living donors (rate of ABMR within 3 months after transplantation is 7.7% compared to 41.2% in patients receiving only plasma exchange) (259). However, the authors showed no differences between the treated and control groups in the incidence of chronic ABMR and death-censored graft survival, suggesting that the blockage of more proximal elements of the complement system may be pivotal in preventing the progression to chronic allograft disfunction (259).

Complement inhibition with Eculizumab to prevent IRI and DGF is still under investigation. In a single-center randomized controlled trial (RCT) of 57 children receiving a single dose of Eculizumab (700 mg/m^2^) prior to transplantation, Eculizumab-treated patients had a significantly better early graft function, less arteriolar hyalinosis, and chronic glomerulopathy on protocol biopsies taken on day 30, 1 year, and 3 years after transplantation; however, an increased number of early graft losses due to flu-like infection has been documented (260).

Other complement-blocking agents have been used in kidney transplantation, and there are ongoing clinical trials evaluating the efficacy of recombinant C1-INH in preventing the development of IRI and DGF, as well as in the prevention and treatment of ABMR (258). C1-INH inhibits both the CP and LP of complement activation during IRI, reducing the release of renal microvesicles by inhibiting the kallikrein-kinin system, and inhibits the coagulation pathway and, consequently, the formation of microthrombi in renal vessels (84, 254, 261). C1-INH is already licensed in many countries for the prevention and treatment of relapse of hereditary angioedema with important results and safety. However, its usage has been extended in other settings in order to prevent the acute development of organ disease and progression to chronic condition, particularly in the setting of kidney transplantation. Vo et al. conducted a phase I/II RCT evaluating the role of C1-INH in preventing ABMR in 20 highly HLA-sensitized recipients (262). After desensibilization, patients randomly received C1-INH 20 IU/kg or placebo intraoperatively and then another seven additional doses in the first month: DGF developed only in one patient in the treatment group, while four patients in the placebo group developed DGF (262). Moreover, no C1-INH-treated patients developed ABMR within 1 month; serum C4 levels recovered more quickly in the study group, and C3 and C4 levels were significantly higher, suggesting that C1-INH treatment may be effective in reducing antigen presentation and DSA production in this setting (262). The same investigators conducted a double-blind RCT where 70 high-risk and/or DCD donor kidney recipients were randomized to receive C1-INH 50 IU/kg intraoperatively and 24 h later versus placebo (263). The development of DGF (need of dialysis in the first post-transplantation week) was reduced in the study group but not statistically significant (42.9 versus 60% in the placebo group) (264); however, dialysis requirement and the mean number of dialysis sessions were reduced in C1-INH-treated group, particularly among recipients of grafts with KDPI. Furthermore, eGFR at 1 year was significantly higher in the treated group compared to the control group (*p* = 0.006), suggesting that C1-INH treatment safely reduces the need for dialysis and prevents progression to chronic graft disfunction (263).

The potential beneficial effect of C1-INH has been recently investigated in two recent studies. In a double-blind RCT performed in 18 DSA-positive recipients with an episode of biopsy-proven ABMR, randomized to placebo or C1-INH treatment in addition to alternate day plasmapheresis and IVIG, Montgomery et al. showed that there was no difference between groups with respect to the primary end points of 20-day graft survival or histological findings, but the C1-INH group showed a sustained improvement in renal function (264). Moreover, transplant glomerulopathy in 14 patients with available allograft biopsies at 6 months was significantly higher in the placebo group (3 of 7 patients) compared to none who received C1-INH (264). Viglietti et al. investigated the usage of C1-INH in six patients with acute ABMR and allograft disfunction that was refractory to standard therapy (steroids, plasmapheresis, high-dose IVIg, and rituximab). The authors showed a significant improvement in renal function (eGFR at 6 months) and, interestingly, a decrease in C1q binding anti-HLA DSA and the proportion of patients with C4d staining in peritubular capillaries in the treated patients: however, no differences in the typical histological findings of ABMR were described (265).

Overall, the main limitation in these studies using complement blockers is the small sample size; moreover, there is no direct competing trial with the use of eculizumab versus C1-INH; therefore, comparison of efficacy of different inhibitors of the complement system in clinical settings is not yet available. Several clinical trials evaluating C1-INH are currently ongoing and will guarantee a better understanding of the opportunities for the use of these agents in clinical transplantation.

In addition to eculizumab and C1-INH, other complement inhibitors have been studied in the transplantation setting, mostly in preclinical studies. These included engineered forms of complement receptor type 1 (CR1) (14), like TP-10 and Mirococept, and synthetic inhibitors of complement convertases (Compstatin) (266). TP-10 has been evaluated to reduce IRI in lung transplantation, showing reduced time of extubation, ventilatory days, and intensive care unit stay compared to patients in the placebo group (267). Furthermore, in a humanized mouse model of islet allograft, pretreatment with Mirococept reduced significantly intraislet inflammation, preserving insulin production by beta cells (268). The EMPIRIKAL trial is ongoing to evaluate the efficacy of an *ex vivo* administered complement inhibitor (Mirococept) in preventing DGF in cadaveric human renal transplantation (269). Pegcetacoplan (APL-2) is a pegylated Compstatin analog that acts as a cyclic peptide inhibitor of C3 and prevents both intravascular and extravascular hemolysis in patients with PHN. In a phase II clinical trial, APL-2 showed significant reduction in lactate dehydrogenas (LDH), total bilirubin, and absolute reticulocyte count with a sustained increase in hemoglobin (270). A phase III study (NCT03500549) comparing eculizumab and APL-2 in patients with PHN is ongoing. Finally, CCX168 (Avacopan), a selective C5a receptor inhibitor, has been investigated in preclinical and clinical studies in patients with ANCA-associated vasculitis. The important advantage of Avacopan is the preservation of the final common pathway of complement activation (MAC); thus, the innate immune response toward microbial agents remains fully active. Results from two phase II clinical trial CLEAR (NCT01363388) and CLASSIC (NCT0222155) showed that Avacopan is safe and effective in patients with ANCA-associated vasculitis allowing a safe reduction or suspension of corticosteroids (95, 271). Preliminary reports from the pivotal phase III ADVOCATE clinical trial (NCT02994927) showed the superiority of Avacopan in terms of sustained remission at 52 weeks and improvement in renal function in patients with ANCA-associated vasculitis in order to replace oral glucocorticoids (95). In this scenario, this therapeutic approach could represent an interesting alternative option also in other complement-based settings, such as IRI in transplantation.

The role of complement in heath and disease as an important component of the antimicrobial defense system raises questions about the safety and feasibility of complement inhibitors. The clinical experience with extended use of these drugs showed that they are considered safe and effective options with limited risk for complications (as infusion-related effects, developing immunogenicity or severe infections): in this scenario, prophylactic measures (meningococcal vaccination before the use of eculizumab or vaccines for other bacteria such as pneumococci) and the prompt antibiotic treatment upon initial signs of infection may minimize the onset of severe adverse effects (272).

## Conclusion and Future Prospective

In summary, a growing body of experimental evidence indicates that complement activation contributes to the pathogenesis of renal inflammaging, particularly in the context of AKI-to-CKD transition. Complement components may regulate a wide range of molecular mechanisms both on infiltrating cells and renal parenchymal cells including scar-forming myofibroblasts, pericytes endothelial, and smooth muscle cells. We provided evidence supporting the pathogenic role of the complement system in promoting tubular epithelial cells senescence by genetic, epigenetic, and protein changes. Cellular senescence and the development of a SASP are involved in the progression from AKI to CKD, leading to common final signaling pathways involved in renal aging and fibrosis.

Currently, there are no validated therapeutic strategies to prevent renal inflammaging. However, promising results in clinical trials using new complement inhibitors suggest that interfering with this pivotal pathway of innate immune system may preserve the kidney from detrimental effect of AKI, reducing the progression of renal fibrosis and the accelerated renal aging.

## Author Contributions

RF and GC mainly contributed to the conception, the design and the writing of the manuscript. AS and MF contributed to writing of the parts relative to: cell-specific effects (for AS) and Complement therapeutics (for MF) and to literature bibliography search. GS and VC supported the final draft editing and revised the manuscript critically for final acceptance for publication. LG and GC supported and supervised the overall design of the article. RF and AS conceived of all figures. RF took the lead in writing the manuscript, reviewers revisions and figure changes. All authors gave final approval for the present version to be submitted.

## Conflict of Interest

The authors declare that the research was conducted in the absence of any commercial or financial relationships that could be construed as a potential conflict of interest.

## Abbreviations

ABMR: antibody-mediated rejection
AKI: acute kidney injury
AP: alternative pathway
C1-INH: C1 esterase inhibitor
CKD: chronic kidney injury
CP: classical pathway
DAMPs: damage-associated molecular patterns
DGF: delay graft function
EndMT: endothelial-to-mesenchymal transition
I/R: ischemia/reperfusion
IRI: ischemia/reperfusion injury
LP: lectin pathway
MAC: membrane attack complex
MASP: MBL-associated serine protease
MBL: mannose-binding lectin
PAI-1/*SERPINE1*: plasminogen activator inhibitor-1
PAMPs: pathogen-associated molecular patterns
PBMC: peripheral blood mononuclear cells
PMT: pericyte-to-myofibroblast transition
PRM: pattern recognition molecules
SASP: senescence-associated secretory phenotype.
