# Corrigendum: Inflammaging and Complement System: A Link Between Acute Kidney Injury and Chronic Graft Damage

**DOI:** 10.3389/fimmu.2020.630855

**Published:** 2021-01-08

**Authors:** Rossana Franzin, Alessandra Stasi, Marco Fiorentino, Giovanni Stallone, Vincenzo Cantaluppi, Loreto Gesualdo, Giuseppe Castellano

**Affiliations:** ^1^ Nephrology, Dialysis and Transplantation Unit, Department of Emergency and Organ Transplantation, University of Bari Aldo Moro, Bari, Italy; ^2^ Department Translational Medicine, University of Piemonte Orientale, Novara, Italy; ^3^ Nephrology, Dialysis and Transplantation Unit, Department of Medical and Surgical Sciences, University of Foggia, Foggia, Italy

**Keywords:** renal aging, complement system, AKI-to-CKD transition, cellular senescence and SASP, complement, inhibition therapy

## Missing Funding

In the original article, we neglected to include the funder the Italian Ministry of Health (AIM 1810057-activity 2 granted to AS; Giovani Ricercatori GR-2009-1608662 granted to GC).

The authors apologize for this error and state that this does not change the scientific conclusions of the article in any way. The original article has been updated.

